# Study on Characteristics of Ultrasound-Assisted Fracture Splitting for AISI 1045 Quenched and Tempered Steel

**DOI:** 10.3390/ma17092143

**Published:** 2024-05-03

**Authors:** Yinfang Jiang, Yangyang Wang, Xiancheng Liu, Deli Sha, Mengcheng Zhu

**Affiliations:** 1 School of Mechanical Engineering, Jiangsu University, Zhenjiang 212000, China; 2212103064@stmail.ujs.edu.cn (Y.W.);; 2 College of Mechanical Engineering, Nantong Institute of Technology, Nantong 226000, China

**Keywords:** con-rod fracture splitting, ultrasonic vibration, ultrasonic amplitude, cracking force, microstructure

## Abstract

Ultrasonic vibration-assisted con-rod fracture splitting (UV-CFS) was used to carry out the fracture experiment of 1045 quenched and tempered steel. The effect of ultrasonic vibration on the fracture properties was studied, the fracture microstructure and the evolution of dislocations near the fracture were analyzed and the microscopic mechanism was analyzed. The results show that in the case of conventional fracture splitting without amplitude, the dimple and the fracture belong to ductile fracture. With the increase in ultrasonic amplitude, the plasticity and pore deformation of the con-rod samples decrease at first and then increase; when the amplitude reaches a certain point, the load required for cracking is reduced to a minimum and the ultrasonic hardening effect is dominant, resulting in a decrease in the plasticity of the sample, a cleavage fracture, a brittle fracture, the minimum pore deformation and high cracking quality. The research results also show that with the increase in ultrasonic amplitude, the fracture dislocation density decreases at first, then increases, and dislocation entanglement and grain breakage appear, then decrease, and multiple dislocation slip trajectories appear. The changes in the dislocation density and microstructure are consistent with the above results.

## 1. Introduction

As the key parts of the engine, the con-rod bears a high thrust load, which has high requirements for its fatigue performance [1], which has high requirements for its fatigue performance. In order to improve the strength and anti-fatigue performance of the con-rod, there are higher requirements for its materials and processing methods [2]. The traditional con-rod machining requires the grinding, broaching and milling of the connecting surface of the con-rod cover and the con-rod body and then carries on the matching work between the con-rod cover and the con-rod body, which is tedious and costly.

Compared with the traditional method, con-rod fracture splitting (CFS) [3] has significant advantages; it fundamentally changes the con-rod production process, which can reduce the con-rod manufacturing process, reduce the investment in equipment and tools and save energy [4]. As a result, the production cost is greatly reduced, the product quality and bearing capacity are improved and a route of high quality, high precision and low cost is provided for the production of the con-rod [5].

At present, with con-rod fracture splitting, it is easy to produce quality defects on the fracture surface and serious deformation of the crank bore in the fracture process [6], so it puts forward higher requirements for the materials used for fracture splitting, requiring the materials to have the characteristic of brittle fracture at room temperature, which seriously limits the selection of cracking materials [7]. At present, the main cracking materials are powder metallurgy, high carbon steel, nodular cast iron and malleable cast iron [8], but the cost of powder metallurgy and high carbon steel is high, and the mechanical properties of cast iron make it difficult to meet the requirements of the con-rod, which hinders the development of fracture splitting.

Quenched and tempered 1045 steel has characteristics of low cost, high strength and good toughness, which meets the working environment of alternating the composite of the con-rod, and is very suitable for the manufacturing material of the con-rod [9]. However, the cracking force of quenched and tempered 1045 steel is too large, which makes it easy to produce large fracture deformation and poor cracking quality, so it is necessary to improve the cracking process of the con-rod [10].

Ultrasound has special potential to improve product quality, reduce production cost and improve production efficiency. At present, ultrasound is widely used in many important fields, such as machining, material forming and preparation, chemistry, medicine and health, textiles, energy saving and environmental protection, bioengineering and so on. The authors of [11,12] used Longitudinal–Torsional Ultrasonic-Assisted Milling (LTUAM) to process Ti-Al6-4V and successfully fabricated micro-dimpled surface textures on Ti-Al6-4V; compared with Conventional Milling (CM), a noticeable decrease in the cutting force was observed in LTUAM. Ni et al. [13] investigated the effect of ultrasonic vibration-assisted milling (UVAM) and minimum quantity lubrication (MQL) on the machining performance of TC4 alloy; the results show that cutting force features could be significantly affected under the UVAM and UVAM&MQL condition compared with that in general milling, and the uniform micro-textured surfaces with improved profile fluctuations could be obtained when applying the UVAM&MQL strategy.

Some scholars have found that the application of ultrasonic vibration in tensile tests of materials such as zinc, magnesium and nickel promotes grain refinement, improves material plasticity and reduces both yield stress and flow stress [14,15]. Storck et al. [16] found that ultrasonic vibration has both acoustic softening and hardening effects on materials. Wen et al. [17] discovered that when the applied ultrasonic amplitude is small, the softening effect predominates, leading to increased material plasticity, while at larger amplitudes, the hardening effect becomes dominant. Because the excessive amplitude leads to the early fracture of the material, and the plasticity of the material decreases to a certain extent, at this time, the hardening effect is dominant. And with the increase in amplitude, the deformation resistance of materials decreases at first and then increases [18]. When the amplitude reaches a certain value, the softening effect of the material is the most obvious, and the fracture is cup-cone-shaped with obvious necking, which is a typical ductile fracture. But with the continuous increase in the amplitude, the deformation resistance of the material increases and the elongation decreases. When the amplitude increases to a certain value, the section of the material changes to brittle transgranular fracture [19].

Although many people have conducted a series of studies on ultrasound-assisted technology, no one has applied ultrasound to CFS. Therefore, based on the new technology of ultrasonic vibration-assisted con-rod fracture splitting (UV-CFS), the effect of ultrasonic amplitude on the behavior of con-rod fracture splitting is studied systematically for the first time in this paper. Through the characterization of crack propagation and the cracking force, fracture morphology and dislocation structure of the con-rod, it is found that the reasonable combination of the acoustic “softening” and acoustic “hardening” effects of ultrasonic vibration can effectively reduce the cracking force and improve the brittleness of the material. In this study, ultrasonic vibration is introduced into con-rod fracture splitting for the first time, which reduces the harshness of the con-rod cracking material and provides a promising auxiliary process for con-rod fracture splitting.

## 2. Materials and Methods

### 2.1. Materials and Devices

The specimen material selected for this paper is tempered 1045 steel, whose chemical composition is shown in Table 1, and the mechanical parameter table is shown in Table 2.

**Figure 1:**
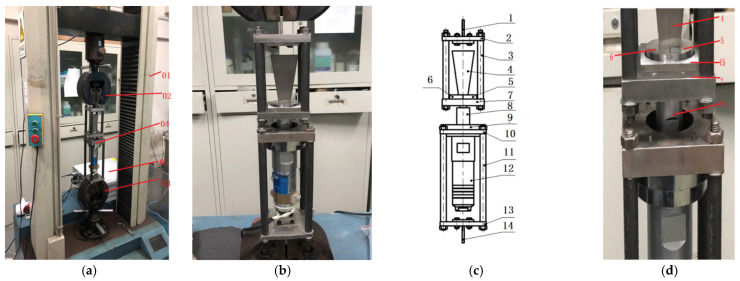
(**a**) Ultrasonic-assisted fracture splitting stretcher, (**b**) cracking device, (**c**) schematic diagram of cracking unit, (**d**) installation of cracking sample. 01. Stretcher, 02. upper chuck, 03. lower chuck, 04. cracking unit, 05. ultrasonic power supply; 1. upper T-shaped block (clamping), 2. upper T-block fixing plate, 3. pull up rod, 4. wedge tool head, 5. right bulging sleeve, 6. left bulging sleeve, 7. sample placement plate, 8. horn, 9. horn fixed circular plate, 10. horn fixed square plate, 11. pull rod, 12. transducer, 13. lower T-block fixing plate, 14. lower T-shaped block (clamping), 15. sample.

The ultrasonic-assisted fracture splitting stretcher, as shown in Figure 1, consists of a stretcher, an ultrasonic-assisted fracture splitting machine and an ultrasonic power supply. The cracking device is mainly composed of an upper T-block, upper T-block fixed plate, upper pull rod, wedge tool head, right expansion sleeve, left expansion sleeve, sample placement plate, horn, horn fixed circular plate, horn fixed square plate, lower pull rod, transducer, lower T-block fixed plate, lower T-block and ultrasonic power supply.

The size of the wedge-shaped tool head was designed according to the half-wavelength theory; the length of the wedge-shaped tool head is about half the wavelength of ultrasonic wave propagation in 1045 steel. The wedge angle of the wedge-shaped tool head is designed to be 16°, the width of the smaller end face is 15 mm, the length between the upper and lower end faces is 135 mm and the thickness is 10 mm. The modal analysis of the tool head was carried out by using the Abaqus finite element analysis software to verify the size of the designed tool head to ensure that when the wedge tool head was connected with the horn, the stress amplitude at the small end of the wedge-shaped tool head was the maximum. The wedge-shaped tool head and the small end of the horn are closely connected and are fixed by M8 × 20 fine thread bolts.

**Figure 2:**
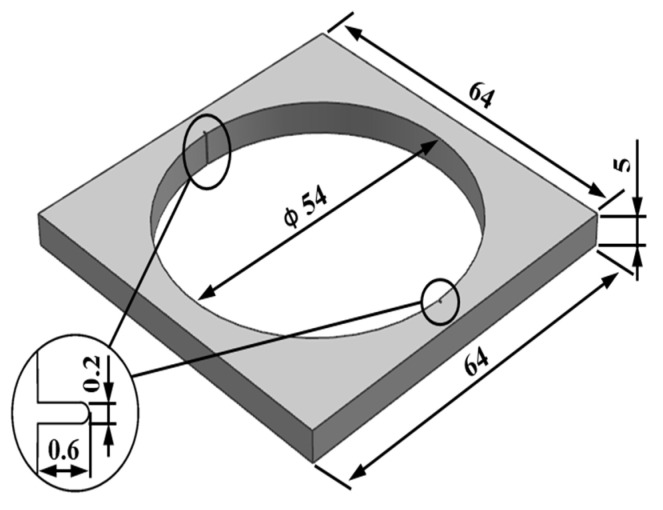
Cracking sample (unit: mm).

**Figure 3:**
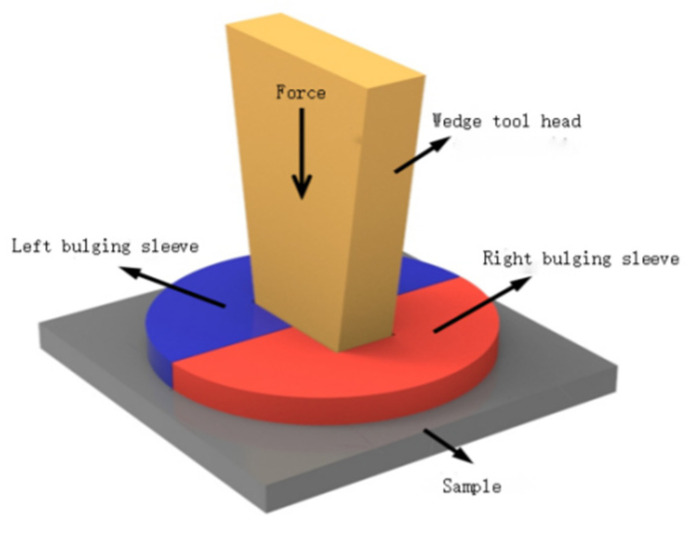
Schematic diagram of ultrasonic vibration-assisted cracking.

The 1045 steel [20] used in the sample is quenched in brine at 840 °C and tempered for two hours at 520 °C. The hardness after heat treatment is around HRC 26. The cracking sample is shown in Figure 2. The crack groove is machined by a laser, the groove depth is 0.6 mm and the groove width is 0.2 mm. Ultrasonic vibration-assisted fracture splitting is shown in Figure 3. The stretcher adopts a microcomputer to control Electro-mechanical Universal Testing Machines (WDW-2000). The ZJS-2000 ultrasonic generator and piezoelectric ceramic transducer are adopted, and the vibration frequency is 20 KHz. Ultrasonic vibration is transmitted to the expansion sleeve through the wedge tool head and then to the sample.

### 2.2. Cracking Force

**Figure 4:**
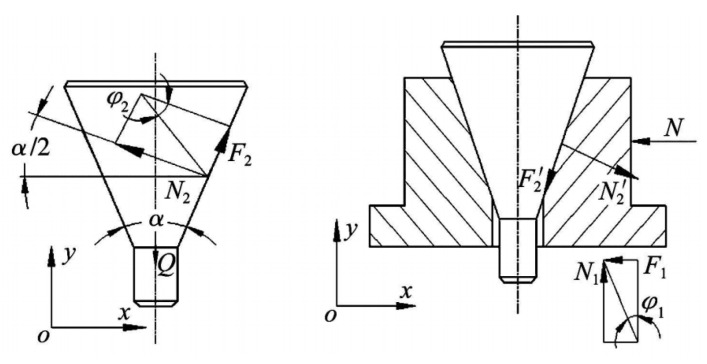
Schematic diagrams of force analysis of cracking unit.

The schematic diagram of the force analysis of the cracking unit is shown in Figure 4, the wedge-shaped tool head is treated as an isolator and the equilibrium equations for forces in the x and y directions are presented:

(1) $$\sum F_{y}=0,\;Q=2(N_{2}sin\frac{\alpha }{2}+F_{2}cos\frac{\alpha }{2})$$

The friction angle of the contact surface between the bulging sleeve and the supporting platform is $\varphi_{1}$, then there are the following:

(2) $$\frac{F_{1}}{N_{1}}=tan\varphi_{1}=\mu_{1}$$

Let the friction angle of the contact surface between the wedge tool head and the bulging sleeve be $\varphi_{2}$, then there are the following:

(3) $$\frac{F_{2}}{N_{2}}=tan\varphi_{2}=\mu_{2}$$

Treat the bulging sleeve as an isolated body, and then analyze the forces separately, and list the equilibrium equations for the forces in the *x*, *y* directions:

(4) $$\sum F_{x}=0,\;N={N_{2}}^{\prime }cos\frac{\alpha }{2}+{F_{2}}^{\prime }sin\frac{\alpha }{2}-F_{1}$$

(5) $$\sum F_{y}=0,\;N_{1}={N_{2}}^{\prime }sin\frac{\alpha }{2}+{F_{2}}^{\prime }cos\frac{\alpha }{2}$$

The ratio *i_p_* of the cracking force *N* to the drawing force *Q* of the stretcher is obtained by combining and solving Formulas (1)–(5):

(6) $$i_{p}=\frac{cos\frac{\alpha }{2}+tan\varphi_{2}sin\frac{\alpha }{2}-tan\varphi_{1}sin\frac{\alpha }{2}-tan\varphi_{1}tan\varphi_{2}cos\frac{\alpha }{2}}{2sin\frac{\alpha }{2}+2tan\varphi_{1}cos\frac{\alpha }{2}}$$

In the above formula, *Q* is the pulling force of the stretcher on the wedge-shaped rod, *N* is the cracking force, *N*_1_ and *N*_2_ are the pressure on the expanding sleeve and the wedge-shaped rod, respectively, *F*_1_ and *F*_2_ are the friction force, α is the angle of the wedge-shaped rod and $\mu_{1}$ and $\mu_{2}$ are the corresponding friction coefficients, respectively. 

### 2.3. Characterization Methods

The fracture morphology of the sample was observed by a JSM-7800F field emission scanning electron microscope of Nippon Electronics Corporation (Beijing, China). The deformation degree of the sample was measured by a 19JPC universal toolmaker’s microscope. Each sample was measured 3 times, and the average value was taken. The XRD was detected by a Japanese ultra X TTR III X-ray diffractometer, the accelerating voltage was 48 kV, the current was 100 mA, the scanning step was 0.02° and the range of the 2θ angle was 32–88°. TEM transmission was performed using a Nippon Electronics Corporation (Beijing, China) high-resolution transmission electron microscope with an accelerating voltage of 200 KV, a point resolution of 0.24 nm, tilt angle of samples: ≥±35°.

**Figure 5:**
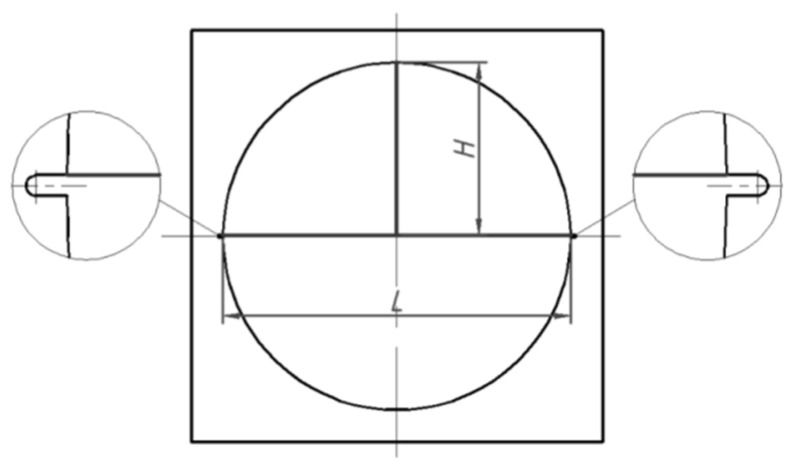
Definition of deformation.

In this experiment, the amplitude is controlled by adjusting the gear position of the ultrasonic generator, adjusting the ultrasonic amplitude to 0 μm, 6 μm, 15 μm, 25 μm and 30 μm, respectively, with 3 samples in each group. Initiate the tensile machine and apply tension until the specimen fractures completely at a fracture speed of 20 mm/min. Record the obtained tension and displacement data, and calculate the fracture force value using Equation (2). After fracture completion, document the deformation in the parallel fracture force direction of the specimen at different amplitudes (change in L before and after fracture) and the deformation in the perpendicular fracture force direction (change in H before and after fracture), as shown in Figure 5.

## 3. Results

### 3.1. Effect of Ultrasonic Amplitude on Cracking Force

**Figure 6:**
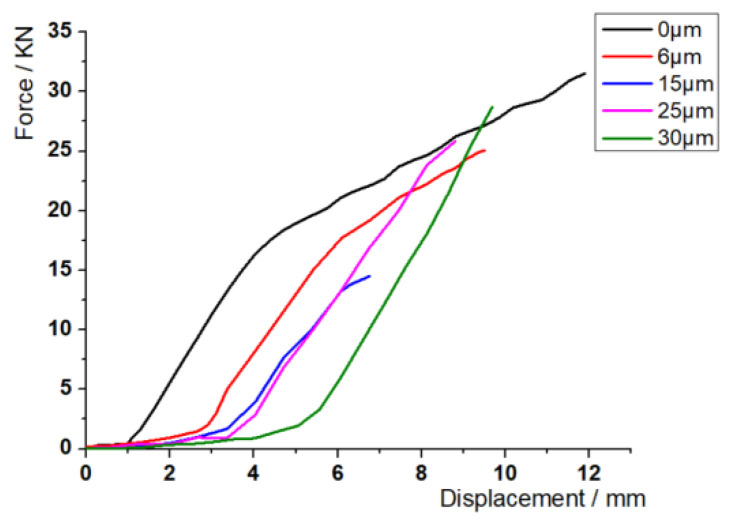
Force–displacement curves.

**Figure 7:**
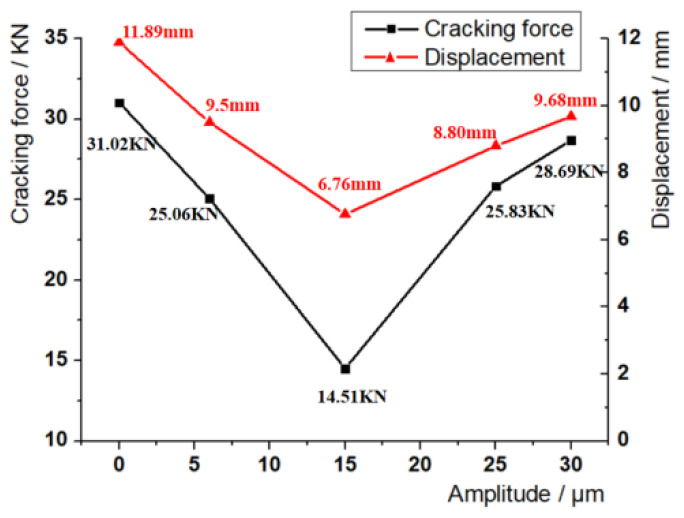
Amplitude–cracking force and amplitude–displacement curve.

The variation curve of the cracking force of the sample and the displacement of the stretcher with different amplitude is shown in Figure 6. At the beginning of tension, the cracking force of the sample with ultrasonic amplitude decreased greatly compared with the sample without amplitude under the same displacement, and the decrease was more obvious with the increase in amplitude; when the sample was at low amplitude (6 μm, 15 μm), the cracking force at fracture decreased compared with that without amplitude, and when the sample was at high amplitude (25 μm, 30 μm), the cracking force at fracture was greater than that without amplitude. The deformation of the sample with ultrasonic vibration-assisted cracking is smaller than that without vibration. When the amplitude is 15 μm, the deformation of the sample at fracture is the smallest, and the load required for cracking is reduced by 53.2%, and the displacement of the stretcher is reduced by 43.1%. With the increase in amplitude, the cracking force and the displacement of the stretcher show a trend of decreasing at first and then increasing. In short, ultrasonic vibration can effectively delay the increase in the cracking force (Figure 7) and reduce the displacement of the stretcher; low-amplitude ultrasound can also reduce the cracking force; there may be an amplitude parameter most suitable for cracking near the amplitude of 15 μm.

### 3.2. Effect of Ultrasonic Amplitude on Hole Deformation

#### 3.2.1. Macro-Deformation at the Cracking Section

**Figure 8:**
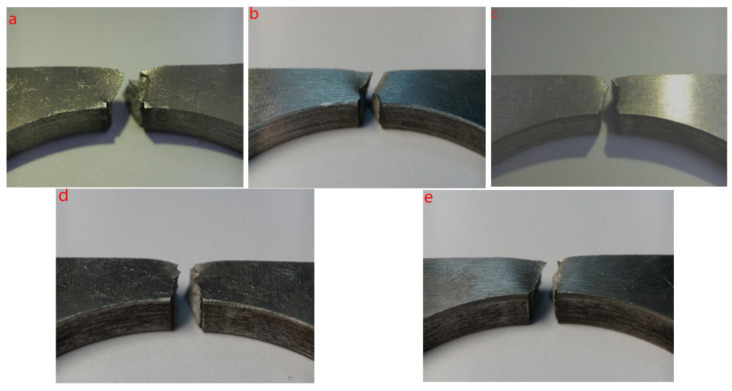
Macrograph of side and fracture after splitting of (**a**) 0 μm, (**b**) 6 μm, (**c**) 15 μm, (**d**) 25 μm, (**e**) 30 μm.

Figure 8 shows the side macrograph of the cracking fracture at different amplitudes of 0 μm, 6 μm, 15 μm, 25 μm and 30 μm. The deformation of the fracture surface of the ordinary non-amplitude cracking sample is large, and there is necking. When the amplitude increases to 6 μm, the fracture necking phenomenon is more obvious, and there is an obvious 45° shear lip on the right side of the fracture surface. It shows that ultrasonic “softening” plays a dominant role when the amplitude is 6 μm. When the amplitude increases to 15 μm, the fracture surface is flat and the necking phenomenon disappears, indicating that “acoustic hardening” is dominant. When the amplitude continues to increase to 25 μm, the necking phenomenon begins to appear again from the side of the fracture, and the fracture surface is still flat. When the amplitude increases to 30 μm, the necking phenomenon is more obvious from the side of the fracture. It can be seen that the increase in ultrasonic amplitude will lead to the necking phenomenon of the fracture surface first enhanced, then weakened and then strengthened. When the amplitude is near 6 μm, “acoustic softening” is dominant, and the material hardens gradually with the increase in the amplitude. There is a threshold or turning point near the amplitude of 15 μm, and the material softens gradually when the amplitude is higher than this threshold.

#### 3.2.2. Hole Deformation after Cracking

**Figure 9:**
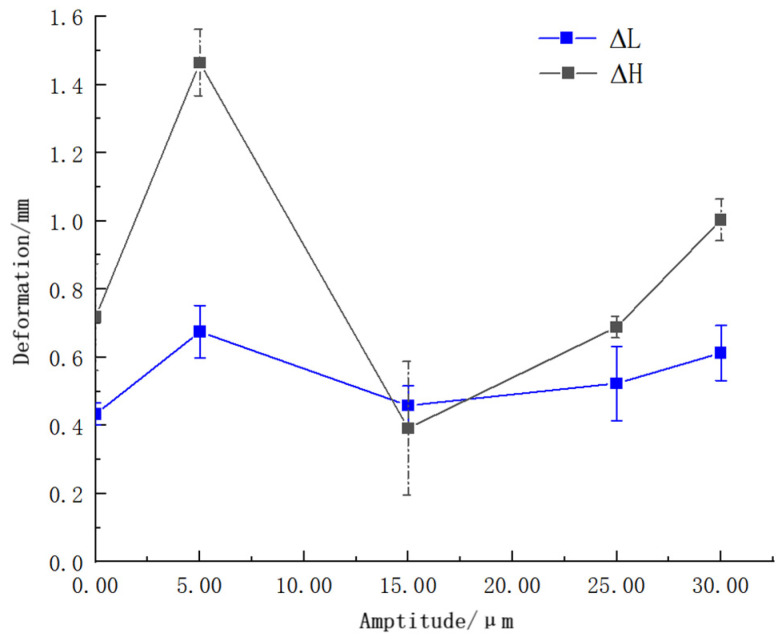
Trend curve of hole deformation of samples under different amplitude.

Measured by the universal toolmaker’s microscope, the results of hole deformation after cracking are shown in Table 3, and the trend curve of hole deformation under different amplitudes is shown in Figure 9. It can be seen from Figure 9 that the change in amplitude has little effect on (L/2) and has a great influence on H. The high-frequency vibration assistant can not necessarily reduce the deformation of the crank bore, and when the amplitude is 6 μm, the deformation of the sample increases sharply, which is significantly higher than that of the conventional non-vibration cracking, which may be because that the low-amplitude high-frequency vibration improves the plasticity of the sample, so that the “softening effect” plays a leading role. When the amplitude is 15 μm, the deformation in the H and L direction decreases, and the cracking quality is improved. The smallest deformation is produced on samples, and there may be a range of amplitude so that the “hardening effect” is dominant, which is more suitable for the cracking of the con-rod. When the amplitude is higher than 15 μm, the deformation of the crank bore increases continuously with the increase in the amplitude, even larger than that of the conventional cracking, which may be due to the transformation of more ultrasonic energy from high-frequency mechanical vibration to thermal energy transfers to the con-rod sample, which in turn improves the plasticity of the material.

### 3.3. Effect of Ultrasonic Amplitude on Microstructure

**Figure 10:**
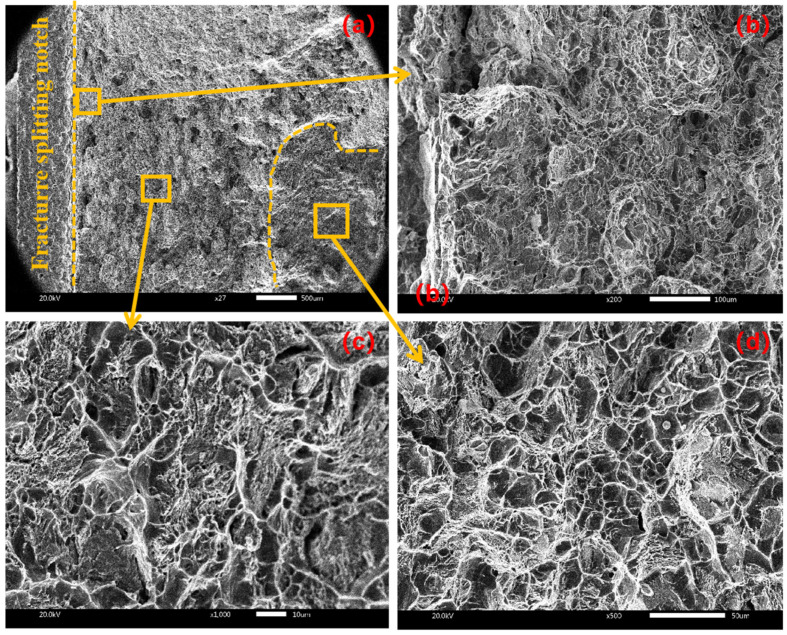
Fracture morphology at amplitude of 0 μm with (**a**) macroscopic fracture, (**b**) crack initiation area, (**c**) extension area, (**d**) termination area.

Divide the macro-fracture into three regions: the initiation region, extension region and termination region. Figure 10 shows the fracture morphology of the sample under conventional cracking without amplitude. From the micrograph of each area of the fracture (Figure 10b–d), there are a large number of dimples in each area, so it can be inferred that the fracture form under no amplitude is mainly ductile fracture.

**Figure 11:**
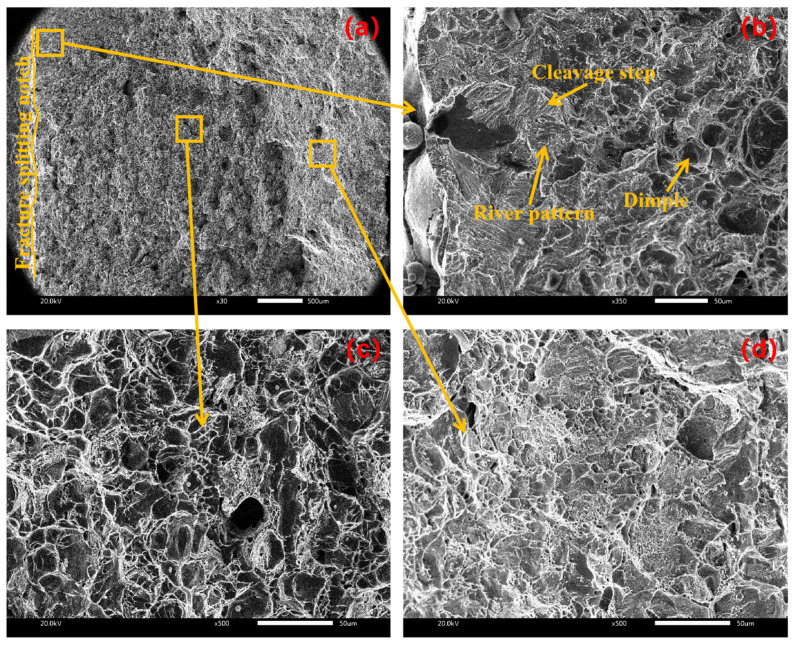
Fracture morphology at amplitude of 6 μm with (**a**) macroscopic fracture, (**b**) crack initiation area, (**c**) extension area, (**d**) termination area.

Figure 11 shows the fracture morphology of the sample when the amplitude is 6 μm. From the crack initiation area in Figure 11b, there are river patterns and cleavage steps near the fracture splitting notch, which belong to cleavage fracture. This is mainly because the ultrasonic vibration and stretcher formed stress superposition near the fracture splitting notch in the initiation area, which accelerated crack propagation in the initiation area and formed cleavage brittle fracture. There are a large number of dimples in the expansion area of Figure 11c and the termination area of Figure 11d, which belong to ductile fracture, from which it is inferred that cleavage fracture and ductile fracture coexist under the amplitude of 6 μm.

**Figure 12:**
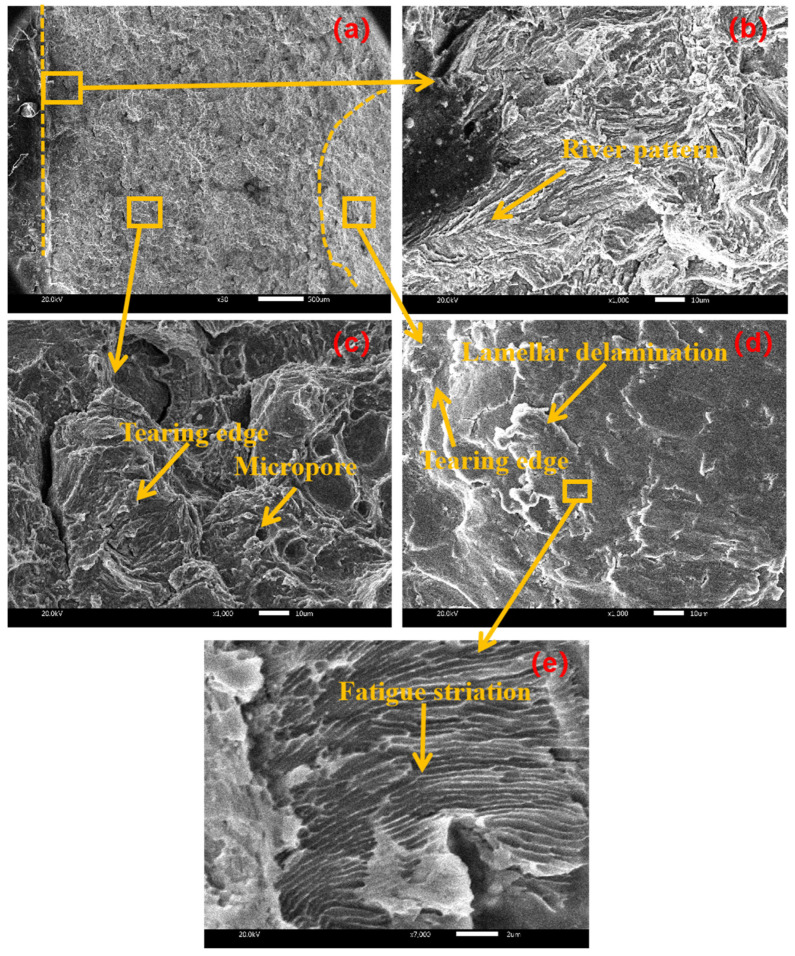
Fracture morphology at amplitude of 15 μm with (**a**) macroscopic fracture, (**b**) crack initiation area, (**c**) extension area, (**d**) termination area (**e**) local magnification.

Figure 12 shows the fracture morphology of the sample with an amplitude of 15 μm. From the initiation area of Figure 12b, there are a large number of river patterns, which belong to cleavage fracture. From the expansion area of Figure 12c, there are many micropores and tearing edges, and the tearing edges are discontinuous, which belong to quasi-cleavage fracture. From the termination area in Figure 12d, there are lamellar delamination and a large number of short and curved tearing edges, which belong to quasi-cleavage fracture; there are also slip marks, and the slip line reflects the path of crack propagation to some extent. It is inferred that when the amplitude increases to 15 μm, the brittleness of the specimen increases, the direction of crack propagation increases and the transition from ductile fracture to cleavage fracture occurs.

**Figure 13:**
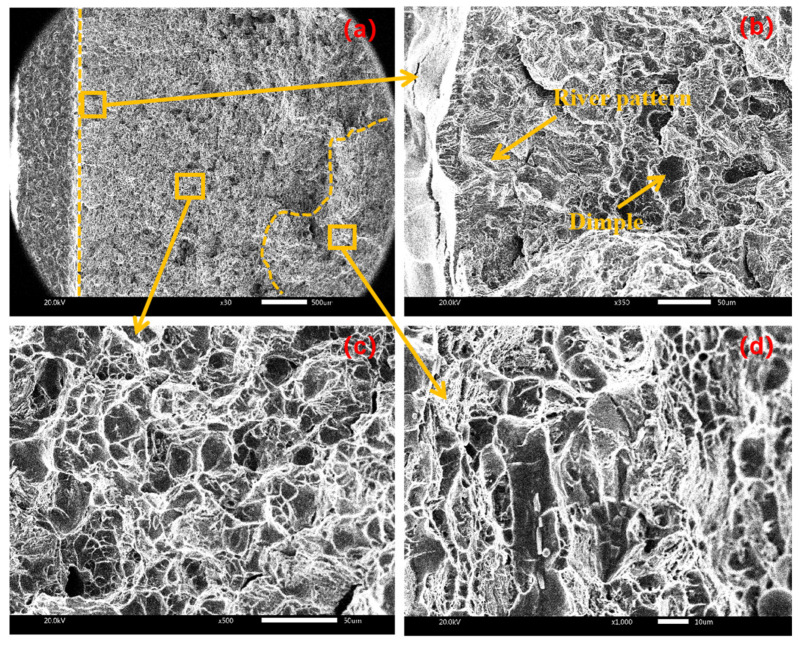
Fracture morphology at amplitude of 25 μm with (**a**) macroscopic fracture, (**b**) crack initiation area, (**c**) extension area, (**d**) termination area.

Figure 13 shows the fracture morphology of the sample with amplitude 25 μm, which is similar to that of the sample with amplitude 6 μm. In Figure 13b, there are a large number of river flowers on the left side of the initiation area and dimples on the right side, indicating that the left side of the initiation area belongs to cleavage fracture and the right side belongs to ductile fracture; there are a large number of dimples in the expansion area of Figure 13c and the termination area of Figure 13d, which belong to ductile fracture. It is inferred that when the amplitude increases to 25 μm, the brittleness of the sample decreases and the plasticity increases.

**Figure 14:**
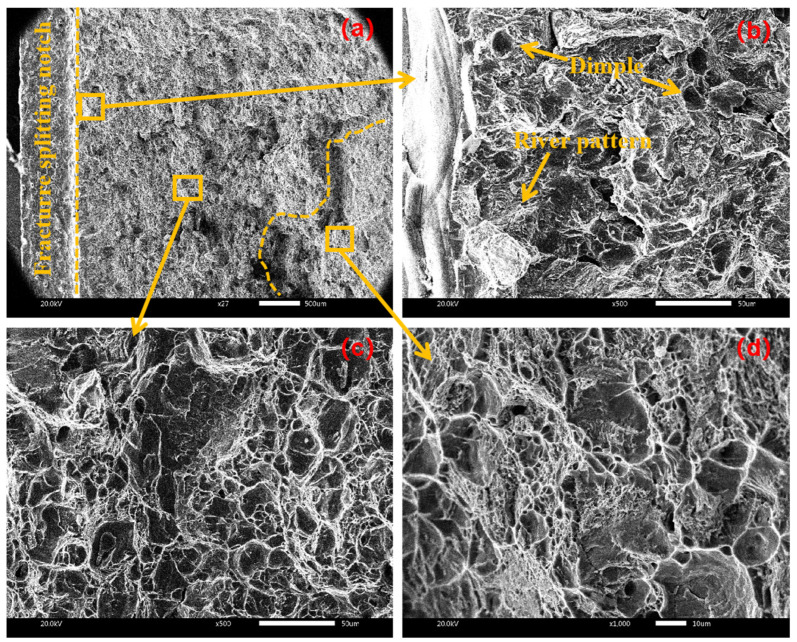
Fracture morphology at amplitude of 30 μm with (**a**) macroscopic fracture, (**b**) crack initiation area, (**c**) extension area, (**d**) termination area.

Figure 14 shows the fracture morphology of the sample when the amplitude is 30 μm. The left side of the initiation area is cleavage fracture, the right side is ductile fracture and there are a large number of dimples existing in the extension area and termination area, which are ductile fracture. It shows that when the amplitude is further increased, the plasticity of the sample is further strengthened.

To sum up, in the case of conventional cracking without amplitude, the dimple characteristic and the fracture form belong to ductile fracture. When the ultrasonic amplitude increases from 6 μm to 30 μm, the plasticity and hole deformation of the con-rod sample decrease at first and then increase. When the amplitude is 15 μm, the fracture form of the con-rod sample is cleavage fracture; at this time, the brittleness is the highest, the hole deformation is the smallest and the cracking quality is the highest.

### 3.4. The Effect of Ultrasonic Amplitude on the Change in Dislocation

From the above research, the ultrasonic amplitude has a great influence on the cracking force, deformation, fracture morphology and ductile–brittle fracture mode of con-rod fracture splitting, so allowing us to better study the microscopic reasons for the above changes during con-rod fracture splitting. The influence of ultrasonic amplitude on the macro-force and deformation of con-rod fracture splitting is studied from the view of the microscopic dislocation motion by the combination of XRD detection analysis and TEM transmission analysis, and the fracture ductile–brittle transfer mechanism is also explained from the microscopic point of view.

#### 3.4.1. XRD Analysis

**Figure 15:**
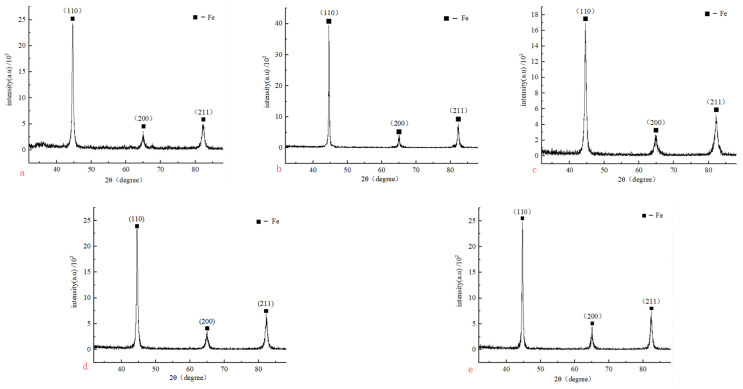
XRD pattern of sample fracture at different amplitude with (**a**) 0 μm, (**b**) 6 μm, (**c**) 15 μm, (**d**) 25 μm, (**e**) 30 μm.

The XRD test of the sample is mainly carried out by the X-ray diffraction experiment on the fracture surface of the cracking sample under different ultrasonic amplitude by an ultra X TTR III X-ray diffractometer. The accelerating voltage is 48 kV, the current is 100 mA, the scanning step is 0.02° and the range of angle 2θ is 32–88°. In Figure 15, we can find that as the ultrasonic amplitude increases from 0 μm to 15 μm and then to 30 μm, the diffraction peaks corresponding to the three crystal planes (110), (200) and (211) show the same law of first enhancement, then weakening and then enhancement, and the FWHM of the diffraction peak decreases at first, then increases and then decreases. This law shows that with the increase in ultrasonic amplitude, the grain size of the cracking material in the cracking area will change to a certain extent, and the grain size will become larger at first, then smaller and then larger. When the amplitude is 0–6 μm, the addition of ultrasound will improve the particle activity of the fracture material, enhance the recovery ability, release the internal stress of the material and increase the grain size to a certain extent. When the amplitude is in the range of 6–15 μm, ultrasound will cause dislocation entanglement and make the grains elongate and break, so that ultrasound can refine the grains to a certain extent, and when the amplitude is higher than 15 μm, the temperature effect caused by ultrasonic amplitude will cause the increase in temperature, resulting in the mutual annexation and growth of grains, then leading to grain enlargement. At the same time, it can also be seen from Figure 15 that the diffraction peaks of (110), (200) and (211) crystal planes keep increasing and decreasing with the increase in ultrasonic amplitude. And there will not be the phenomenon that the peak value of the crystal plane changes greatly while others’ change inversely, indicating that ultrasonic fracture splitting does not affect the grain orientation or the preferred orientation along a certain plane of the cracking material. 

**Figure 16:**
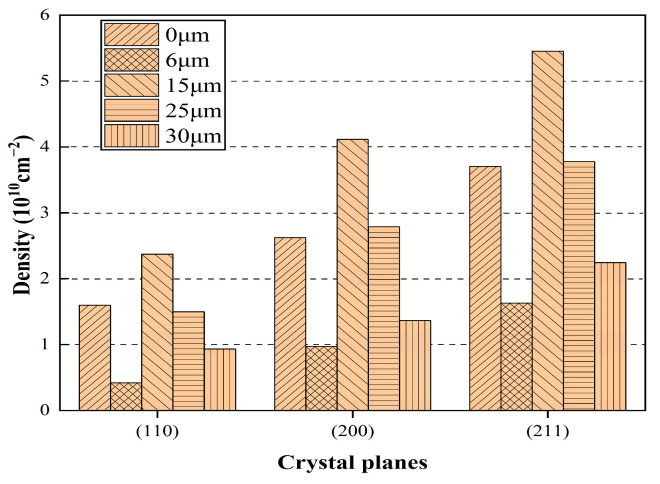
Dislocation density of different crystal planes at different amplitude.

MID Jade 6 diffraction peak processing software was used to calculate the FWHM of the diffraction peak under different amplitude. According to the relationship obtained by Dunn et al. [21,22,23], the dislocation density was obtained. The calculated dislocation densities of different crystal planes are shown in Figure 16.

**Figure 17:**
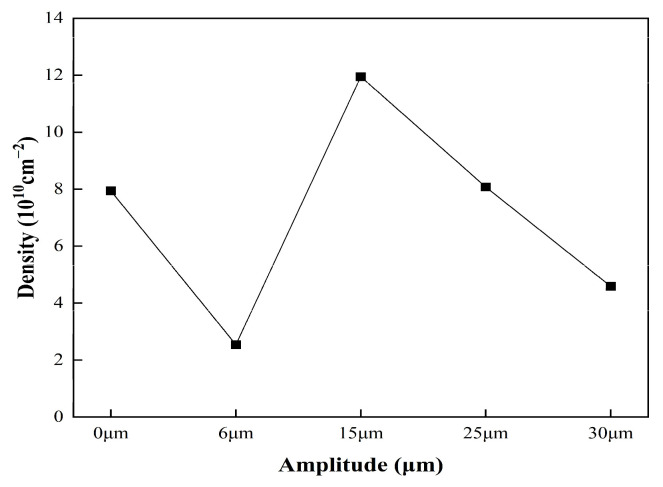
Curves of total dislocation density at different amplitude.

In Figure 16, with the increase in ultrasonic amplitude, the dislocation changes in each crystal plane are complex, and the dislocation density of each crystal plane reaches the order of 1010 cm^−2^. At different amplitude, the dislocation density of the (211) crystal plane is always higher than that of the other two crystal planes, which is mainly due to the maximum tensile stress on the (211) crystal plane and the faster deformation of the material during cold plastic deformation; the dislocation density also increases sharply at the same time; it also increases the resistance of dislocation movement. With the increase in ultrasonic amplitude, the dislocation density of each crystal plane decreases at first, then increases and then decreases. We can add the dislocation densities of (110), (200) and (211) together and find out the change rule of it. The total dislocation density curve under different amplitude is shown in Figure 17.

In Figure 17, the total dislocation density decreases at first and then increases and then decreases with the increase in amplitude. At low amplitude (0–6 μm), ultrasound can improve the particle activity of the con-rod during deformation, release the internal stress, annihilate the dislocation and decrease the dislocation density, and the larger the amplitude is, the more obvious this phenomenon is. When the amplitude is about 15 μm, the energy provided by ultrasound can not only completely release the internal stress of the material but also produce a new dislocation multiplication phenomenon. Due to the higher amplitude, the continuous increase in the amplitude will increase the strain rate of the material at the same time. As a result, the increment rate of dislocations increases, which means that the critical shear stress of the slip system is increased, slip is less likely to occur and the temperature is still in a low state; the material is not easy to recrystallize. These lead to the increase in dislocation density. When the amplitude reaches a larger value (25–30 μm), the increase in amplitude will highlight the temperature effect, resulting in an increase in the temperature of the material during cracking, then resulting in the enhancement of the dynamic characteristics of the atom and the critical shear stress required for the start-up of the slip system decreasing, which reduces the slip resistance between the dislocation motion and the crystal plane [24]. At the same time, the increase in temperature will also make the material more prone to recrystallization. All these will lead to the decrease in dislocation density.

#### 3.4.2. TEM Analysis

**Figure 18:**
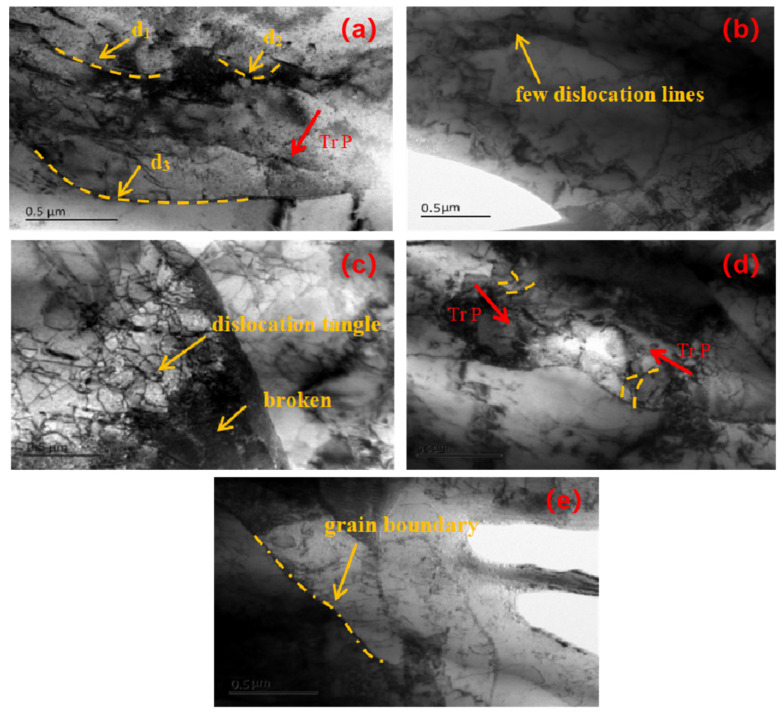
Micrograph of sample observed by TEM with (**a**) 0 μm, (**b**) 6 μm, (**c**) 15 μm, (**d**) 25 μm (**e**) 30 μm.

Because the crack propagation area of the fracture surface always accounts for a large proportion of the fracture area under different amplitude, in order to compare the effect of amplitude on the dislocation of the fracture surface, we observe the change in the dislocation mechanism in the propagation area under different amplitude. The slice specimens in the vertical direction of the fracture crack propagation area were observed by a transmission electron microscope. First of all, the right part of the fracture surface of the cracked sample was cut from the middle of the fracture surface by WEDM to cut a sheet with a thickness of 200 μm and a length of 4 mm perpendicular to the depth of the fracture splitting notch, then the thickness was about 50 μm with metallographic sandpaper, and then the circle of ∅3 mm was punched out along the middle of the sheet on the punching machine, and then the central area of ∅3 mm was thinned to the desired thickness by an ion thinning instrument. Finally, different samples were observed by a transmission electron microscope (TEM). Their micrograph is shown in Figure 18, and the observed area is the region with a depth of 2 mm in the center of the fracture, and the mechanism of amplitude on the microstructure of cracking samples is analyzed.

In Figure 18, when the amplitude is 0 μm and only the tension machine is used for cracking, it belongs to static tension. The matrix material continues to deform in a certain direction after being subjected to the breaking force exerted by the expanding sleeve, so does the dislocation movement, as shown in Figure 18a; multiple dislocation lines and the dislocation slip direction TrP have been marked with a thick arrow. When two of the dislocation lines d1 and d2 meet and act on each other, the dislocation will be pinned. At the same time, the dislocation at the pinning point (usually impurity elements) will be blocked and difficult to move. However, the dislocation lines on both sides of the pinning point continue to move, which causes the dislocation to become arched. When the dislocation motion continues, two dislocation lines are connected. This mechanism forms a new dislocation d3 and produces an immovable dislocation segment [25]. This phenomenon results in the conventional con-rod fracture splitting requiring a larger maximum cracking force; at the same time, the con-rod itself also deforms greatly, which affects the quality of cracking processing. When the amplitude is 6 μm, the addition of ultrasound can effectively weaken the above directivity, improve the activity of particles and release the internal stress of the material, resulting in a certain reduction in dislocation density and the number of dislocation lines, as shown in Figure 18b. When the amplitude is further increased to 15 μm, the dislocation density increases, the dislocation entanglement is obvious and grain fragmentation and a grain size decrease appear at the same time, as shown in Figure 18c, which shows that the cracking fracture belongs to brittle cleavage fracture to a certain extent. When the amplitude is higher than a certain threshold, the thermal effect caused by ultrasound begins to dominate. When the amplitude is 25 μm, the thermal effect caused by ultrasound will trigger the partial recrystallization of the material, coarsening the internal polymerized grain, and constantly thermally activate the dislocation migration at the interface, resulting in dislocation slip; the energy of the system and dislocation density and interface energy continue to decrease. At the same time, this thermal effect is also conducive to the initiation of multi-slip systems, so that dislocations in different grains can cross the grain boundary through dislocation movement, so multiple slip characteristics can be found in the same grain, that is, the dislocations of each slip system move along their respective directions, as shown in Figure 18d. This will lead to the con-rod softening to some extent during cracking, and the fracture tends to ductile fracture. When the amplitude increases to 30 μm, the dislocation density further decreases, and the grain boundaries of recrystallized grains continue to become flat, coarse and obvious, as shown in Figure 18e, which ensures that the material tends to a lower energy state during cracking, thus more severe deformation can occur, and the con-rod tends to ductile fracture during cracking.

In summary, it can be seen from the micrographs of the transmission electron microscope under different amplitude in Figure 18 that the dislocation density at the fracture of the cracking sample decreases when the amplitude ranges from 0 μm to 6 μm, increases when the amplitude ranges from 6 μm to 15 μm and decreases when the amplitude ranges from 15 μm to 30 μm.

## 4. Discussion—An Analysis of the Mechanism of Ultrasound-Assisted Cracking

The results show that when the amplitude increases from 0 μm to 6 μm, low-amplitude vibration will increase the temperature of the crack tip, increase the mobility between atoms, reduce the resistance of the dislocation motion, increase the atomic transition frequency and the vacancy concentration, increase the self-diffusion of atoms in the matrix and the slip ability of dislocations, continuously release the internal stress of the material and the flow stress of the metal decreases obviously, weakening the stress concentration effect. And dislocations are easier to redistribute to form a more stable lattice in energy, which speeds up the mutual destruction of dislocations and decreases the density of dislocations. At the same time, due to the decrease in dislocation density and dislocation motion resistance, the dislocation emission at the crack tip becomes easier during cracking, which leads to the passivation of the crack tip, the improvement in the plasticity of the material and the fracture surface tends to ductile fracture. Finally, the deformation of the sample is intensified after cracking.

When the amplitude increases from 6 μm to 15 μm, due to the high amplitude, ultrasound can not only completely release the internal stress of the material but also cause new internal stress and dislocation density to increase again. At the same time, the mutual accumulation and entanglement of dislocations are caused by the characteristics of high ultrasonic frequency and a different direction of motion. And near the fracture splitting notch, due to the sudden change in the cross-section size of the sample and the large dislocation density, leading to lattice distortion and the force effect of stress field and the atom deviating from its equilibrium position, while the dislocation plug group means that many dislocations are concentrated together, the superposition of the force increases the total force, which will produce a great force near the dislocation plug group, so the stress superposition is concentrated in the dislocation plug group, causing the concentrated distribution of stress in the cracking trough. With the increase in amplitude, the dislocation density increases sharply, and the stress concentration becomes more obvious, making the hardening effect of the material more obvious, which makes the dislocation emission at the crack tip of the material become more difficult, and the plasticity of the material decreases. Thus, the fracture surface tends to brittle fracture and finally reduces the deformation degree of the sample after cracking. The peak value of the hardening stress peak increases with the increase in ultrasonic vibration energy, and the higher the vibration energy is, the more significant the hardening effect is [26]. This is consistent with the research findings of Xie et al. [27], which suggest that the hardening effect predominates when a large amplitude of ultrasonic vibration is applied during tensile testing. When the amplitude reaches 90% A (A is the maximum amplitude of the ultrasonic vibration device), the cross-section of the material is brittle transgranular fracture. When the ultrasonic vibration energy is high, the hardening effect is dominant, and the high vibration energy changes the deformation mechanism of the material, which leads to brittleness [28].

When the amplitude increases from 15 μm to 30 μm, because the ultrasonic amplitude is too large, the limiting effect of sleeve expansion on ultrasonic vibration is prominent, so that more ultrasonic energy is converted into thermal energy, and the thermal effect caused by ultrasound is dominant. During cracking, the temperature of the fracture surface is high, and the higher the amplitude is, the higher the temperature is, which leads to the intensification of dislocation annihilation and the decrease in dislocation density with the increase of in amplitude. At the same time, the thermal effect makes it easier to trigger the partial recrystallization of the material, the coarsening of material polymerization, the activation of the dislocation migration at the interface, the slip phenomenon and the continuous decrease in the dislocation density; these increase the plasticity of the material, lead to the fracture surface tending to ductile fracture and finally aggravate the deformation of the sample after cracking.

Under the premise of non-resonance, the realization of the cracking of the con-rod sample mainly depends on the tension of the stretcher, and the ultrasonic vibration only plays an auxiliary role. The ultrasonic vibration with appropriate amplitude should be selected to better realize the cracking of the con-rod.

The amplitude of high-frequency vibration has a great influence on the force, deformation and fracture of the con-rod in the cracking process. The influence of high-frequency vibration on the material deformation process includes the force effect, thermal effect and so on. On the one hand, due to the superposition of the force, the stress concentration is higher, and the crack appears at the bottom of the cracking tank earlier; on the other hand, after the material absorbs the vibration energy, the temperature and the activity of particles in the material increase, which provides energy for the movement of dislocations, so that the plasticity of the material is also partially improved. There is a possible critical amplitude near 15 μm, which can make the ultrasonic energy act on the con-rod in the form of mechanical vibration efficiently.

By observing the fracture surfaces of the samples with different amplitude, it is found that ultrasonic vibration can improve the brittleness of the root area of the fracture splitting notch, so that the crack appears and propagates at the root of the cracking tank earlier. The high-frequency vibration with an amplitude of 6 μm has little effect on cracking and shows ductile fracture in both the crack propagation area and the rapid fracture area. When the amplitude is 15 μm, the ultrasonic vibration promotes both the crack propagation area and the rapid fracture area, which improves the brittleness of the fracture surface and is more beneficial to the cracking of the con-rod. When the amplitude is 25 μm and 30 μm, the extrusion pressure between the expanding sleeve and the crank bore of the sample limits and weakens the effect of high-amplitude vibration, so that more ultrasonic energy is transferred from high-frequency mechanical vibration to thermal energy to the con-rod sample and then improves the plasticity of the material and makes the fracture surface ductile, which is not conducive to the cracking of the con-rod.

## 5. Conclusions

In order to expand the range of cracking con-rod materials and reduce the requirements of process parameters, ultrasonic-assisted con-rod fracture splitting was studied by using a con-rod material of quenched and tempered 1045 steel. The effects of amplitude on cracking force and deformation, ductile–brittle transition and dislocation change were tested and analyzed; the general law of the effect of different amplitude on cracking was found out, so as to better control the cracking quality. The main conclusions of this paper are as follows:

(1) When the amplitude increases from 0 μm to 15 μm and then to 30 μm, the cracking force and the displacement of the stretcher decrease at first and then increase. At 15 μm, the cracking force of the sample is the smallest and the deformation of the sample is the smallest.
(2) There is a threshold near the amplitude of 15 μm. When the amplitude is lower than this threshold, ultrasonic vibration plays a major role in cracking, and the low-amplitude “softening” and high-amplitude “hardening” effects of ultrasound are consistent with the experimental results.
(3) When the amplitude increases from 0 μm to 6 μm, the dislocation density decreases, the dislocation lines at the center depth of the fracture 2 mm become reduced and the direction of the dislocation movement becomes less obvious. When the amplitude increases from 6 μm to 15 μm, the dislocation density increases, resulting in dislocation entanglement and grain fragmentation. When the amplitude increases from 15 μm to 30 μm, the dislocation density decreases, and multiple dislocation slip trajectories appear.
(4) The increase in amplitude will lead to the decrease in dislocation density at first, then an increase and then a decrease; the plasticity of the fracture surface will increase, then decrease and then increase. The effect of amplitude on dislocation leads to the change in fracture morphology.
(5) There is a threshold near the amplitude of 15 μm. When the amplitude is this threshold, the minimum cracking force and sample deformation can be guaranteed, the fracture surface is smooth without a necking phenomenon and the fracture surface is brittle and flat.

## Figures and Tables

**Table 1:** Chemical composition of 1045 steel (wt%).

C	Si	Mn	P	S	Cr	Ni	Cu
0.42~0.50	0.17~0.37	0.50~0.80	≤0.045	≤0.06~0.07	≤0.25	≤0.30	≤0.25

**Table 2:** Mechanical parameters of 1045 steel.

Material	E/MPa	Poisson’s Ratio (*v*)	Yield Strength $\sigma_{s}$/MPa	Tensile Strength $\sigma_{b}$/MPa	Rupture Stress $\sigma_{f}$/MPa	Rupture Strain $\varepsilon_{f}$/MPa
1045 steel	210	0.3	710	993	1727	0.21545

**Table 3:** Measurement of hole size deformation.

NO.	Amp/μm	Before L/2/mm	After L/2/mm	∆(L/2)/mm	Before H/mm	After H/mm	∆H/mm	Deform/mm
1	0	26.940	26.739	0.201	26.718	27.423	0.705	0.906
2		26.933	26.718	0.215	26.708	27.428	0.720	0.935
3		26.899	26.665	0.234	26.634	27.362	0.728	0.962
4	6	26.960	26.635	0.325	26.817	28.305	1.488	1.813
5		26.863	26.523	0.340	26.713	28.128	1.415	1.755
6		26.930	26.583	0.347	26.655	28.145	1.490	1.837
7	15	26.945	26.727	0.218	26.869	27.255	0.386	0.604
8		26.864	26.632	0.232	26.734	27.130	0.396	0.628
9		26.835	26.597	0.238	26.708	27.101	0.393	0.631
10	25	26.945	26.690	0.255	26.723	27.354	0.631	0.886
11		26.860	26.597	0.263	26.321	27.060	0.739	1.002
12		26.935	26.668	0.267	26.708	27.404	0.696	0.963
13	30	27.028	26.711	0.317	26.302	27.282	0.980	1.297
14		26.946	26.648	0.298	26.450	27.423	0.973	1.271
15		26.867	26.563	0.304	26.653	27.708	1.055	1.359

## Data Availability

The data will be provided as required.
